# *Triportheus albus* Cope, 1872 in the Blackwater, Clearwater, and Whitewater of the Amazon: A Case of Phenotypic Plasticity?

**DOI:** 10.3389/fgene.2017.00114

**Published:** 2017-08-31

**Authors:** José D. A. Araújo, Andrea Ghelfi, Adalberto L. Val

**Affiliations:** ^1^Laboratory of Ecophysiology and Molecular Evolution, National Institute of Amazonian Research Manaus, Brazil; ^2^Federal University of Amazonas Manaus, Brazil; ^3^Kazusa DNA Research Institute Kisarazu, Japan

**Keywords:** Rio Negro, Tapajós River, Solimões River, differential expression, RNA-Seq, acidic pH, ionic regulation

## Abstract

The Amazon basin includes 1000s of bodies of water, that are sorted according to their color in three types: blackwater, clearwater, and whitewater, which significantly differ in terms of their physicochemical parameters. More than 3,000 species of fish live in the rivers of the Amazon, among them, the sardine, *Triportheus albus*, which is one of the few species that inhabit all three types of water. The purpose of our study was to analyze if the gene expression of *T. albus* is determined by the different types of water, that is, if the species presents phenotypic plasticity to live in blackwater, clearwater, and whitewater. Gills of *T. albus* were collected at well-characterized sites for each type of water. Nine cDNA libraries were constructed, three biological replicates of each condition and the RNA was sequenced (RNA-Seq) on the MiSeq^®^ Platform (Illumina^®^). A total of 51.6 million of paired-end reads, and 285,456 transcripts were assembled. Considering the FDR ≤ 0.05 and fold change ≥ 2, 13,754 differentially expressed genes were detected in the three water types. Two mechanisms related to homeostasis were detected in *T. albus* that live in blackwater, when compared to the ones in clearwater and whitewater. The acidic blackwater is a challenging environment for many types of aquatic organisms. The first mechanism is related to the decrease in cellular permeability, highlighting the genes coding for claudin proteins, actn4, itgb3b, DSP, Gap junction protein, and Ca^2+^-ATPase. The second with ionic and acid-base regulation [rhcg1, slc9a6a (NHE), ATP6V0A2, Na^+^/K^+^-ATPase, slc26a4 (pedrin) and slc4a4b]. We suggest *T. albus* is a good species of fish for future studies involving the ionic and acid-base regulation of Amazonian species. We also concluded that, *T. albus*, shows well defined phenotypic plasticity for each water type in the Amazon basin.

## Introduction

The rivers of the Amazon are interconnected to the central channel, the Amazon River, sheltering a rich ichthyofauna and allowing the entire Amazon basin to be linked through its waters. The river connection make possible for species to migrate among the rivers of the region (Val and Almeida-Val, 1995). However, these rivers contain different water types, due to the geographical location of each river and the materials that are deposited on their beds (Sioli, 1984; Konhauser et al., 1994). In many cases, the physicochemical parameters of the waters govern the selection of species which inhabit them (Val and Almeida-Val, 1995). This selection depends on the capacity that species possess to get adapted to the environmental conditions to which they are exposed (Evans et al., 2005; Wood et al., 2007). In this context, phenotypic plasticity has recently gained significance in transcriptome analyses and can be defined as the ability of a genotype to produce variable phenotypes under different environmental conditions. Thus, the phenotypic plasticity supporting physiological adjustments necessary for the transition between environments is valuable for many species (Gibbons et al., 2017).

The waters of the Amazon constitute a singular place to analyze that issue, as water bodies containing blackwater, clearwater, and whitewater (Sioli, 1984) are interconnected.

Indeed, physicochemical parameters are closely related to their characteristic colors. Three major rivers have these water profiles: the Negro River (blackwater), considered one of the most challenging aquatic environments for aquatic species, due to its natural acidic water and low level of ions. The presence of significant concentrations of dissolved organic matter (DOM), produced by plant decomposition because of the seasonal flooding of part of the forest (Sioli, 1984; Ertel et al., 1986), is responsible for releasing humic and fulvic acids in the water, which in turn, have carboxylic groups (-COOH) and hydroxylic (-OH) groups in their structures. These dissociate themselves and release H^+^ ions into the water, thus reducing the pH in the environment (Queiroz et al., 2009). Several studies have been developed in an attempt to understand how a river with such an enormous environmental challenge houses a significant diversity of fish, estimated at, approximately, 1,000 species (Val and Almeida-Val, 1995; Gonzalez et al., 2002; Wood et al., 2014; Duarte et al., 2016).

The Solimões/Amazonas river, the primary representative whitewater, is the largest freshwater river on the planet. It has its distinctive color due to the high quantity of material in suspension derived from sediments from the Andes. These sediments are transported in large volumes along the whole river, being deposited on the banks, forming sandbanks with the clayey material known in the region of the várzea (the area on the riverbanks that gets flooded during the flood season) (Furch, 1984). The Solimões River is richer than the Rio Negro in dissolved ions and its electrical conductivity is the highest one when compared to clearwater and blackwater, with an average of 98.8 μS/cm (Küchler et al., 2000). The predominant ions in its waters are Na^+^, K^+^, Mg^2+^, and Ca^2+^. The pH of whitewater is close to neutrality (6.6) (Sioli, 1984; Gaillardet et al., 1997).

The Tapajós River is known for its crystalline, slightly greenish waters. Its waters contain little clayey sediment, due to the drainage that the river makes on the soil of pre-Cambrian origin (Sioli, 1984). Just as the Negro River, it has areas of igapós (flooded forests) on its banks, depending on the seasonal cycle. The pH is around 6.5 and it has low conductivity (14.4 μS/cm), with a reduced amount of suspended material. Whereas the physicochemical parameters of its waters are referenced to as an intermediary between blackwater and whitewater rivers (Duncan and Fernandes, 2010), the clearwater is the one with the highest annual variation on its physicochemical parameters in the Amazon basin, mainly as regards to the water pH (Junk and Furch, 1980). Therefore, both the Tapajós River and the Solimões River have a pH close to neutrality, while the Negro River has acidic waters (Sioli, 1984).

Depending on the annual water fluctuation (seasonal ebb or river flooding), the physicochemical parameters can be altered. Observing the environmental conditions of different types of waters makes the challenges that these environments impose on ichthyofauna evident (Furch, 1982).

These environments are home to the most diverse ichthyofauna in the world. Many species simultaneously inhabit two of these environments (reviewed by Val and Almeida-Val, 1995). Few, however, have developed biological mechanisms to live simultaneously in the three types of water in the Amazon. Among these, we highlight the species *Triportheus albus*, popularly known in the region as sardine. This species is often found in all three types of water in the Amazon basin (INCT/ADAPTA Project Report 2012–2013). The understanding of how this species responds to the different environmental conditions prevailing in the three types of Amazonian environments was the primary factor that challenged us in the present study.

Considering the environmental and physicochemical characteristics of the waters in which the presence of the *T. albus* occurs, the purpose of our study was to analyze if the gene expression of *T. albus* is determined by the different types of water. Our hypothesis is that *T. albus* presents phenotypic plasticity to live in blackwater, clearwater, and whitewater.

## Materials and Methods

The experimental procedures were approved by the Animal Use Ethics Committee of the Brazilian National Institute for Research of the Amazon (CEUA-INPA) (Protocol 026/2015). The permit for the collection of the biological material to carry out the research was authorized by the Brazilian Institute of Environment and Renewable Natural Resources (IBAMA/SISBio), number 29837-8/2015.

### Collection of Samples

Specimens of *T. albus* were captured in their natural environments, covering the different water types of the Amazon basin in expeditions carried out in July and August 2015. See Supplementary Table S1 for length and mass of the analyzed fish. Blackwater specimens were captured on the banks of the Negro River, in the Anavilhanas Archipelago (2°43′11.8″S, 60°45′19.1″W). Fish collection in whitewater was carried out on the banks of the Solimões River, near the island of Marchantaria (3°14′47.0″S, 59°57′23.3″W). The collection of specimens in clearwater was carried out on the banks of the Tapajós River (2°48′46.3″S, 55°02′21.2″), for information on fish in Supplementary Table S1.

Gills were the analyzed tissue because they are the primary site for osmotic regulation, excretion of ammonia, as well as an important site for gas exchange and regulation of body fluid pH. Thus, nine *T. albus* specimens were captured using fishing line and hook, three individuals for each environmental condition. After captured, they were sacrificed by severing their spinal cord, the gills were removed and immediately stored in RNALater^®^ (Ambion^®^), and kept at 4°C, until arrival at the Laboratory of Ecophysiology and Molecular Evolution (LEEM), at INPA’s facilities in Manaus, Amazonas, Brazil. Water physicochemical parameters were measured at the same collection site of the organisms between 5 and 7 pm. During the period of collection, the seasonal “river flooding” period prevailed. The pH values were measured using a pHmeter (Micronal model B374) and dissolved oxygen and conductivity was measured with an oxygen meter (YSI, model 55/12 FT).

### RNA Extraction and Library Construction

Total RNA extraction was performed individually for each of the nine *T. albus* gill tissue samples using the TRIzol^®^ reagent protocol (Invitrogen^TM^). After that, 30 μL of ultrapure water (Nuclease-Free) was added to each bullet tube containing the RNA extract, which was stored at -80°C until the time of analyses. The quantification of extracted RNA was determined using a BioAnalyzer Agilent 2100 (Agilent Technologie^®^). The procedures for building the libraries were performed according to the TruSeq RNA Sample Prep v2 LS protocol (Illumina^®^), observing the manufacturer’s recommendations.

The mRNA was isolated from the total RNA using magnetic oligo (dT) beads that were bound to the poly (A) tail of the mRNA. Then, the first cDNA strand was synthesized using reverse transcriptase and random primers. The second cDNA chain was immediately synthesized, using the enzymes DNA Polymerase I and RNAse H. It was then added to the end 3′ of the fragments of a single A nucleotide (adenine). The adapters were bound to these fragments, the PCR was then performed for the enrichment of the libraries.

Finally, the quality and quantification of the constructed libraries were analyzed using the BioAnalyzer Agilent 2100 (Agilent Technologie^®^) and Real-Time PCR 7500 (Applied Biosystems^®^). Three biological replicates of samples from each environmental condition were sequenced on the MiSeq^®^ platform (Illumina^®^) in three sequencing runs – a paired-end run (2 × 150 cycles) and two paired-end runs (2 × 250 cycles). The data generated in this study are stored in the National Center for Biotechnology Information/Sequence Read Archive (NCBI-SRA), project number PRJNA391967.

### Quality Control and Reassembly

The bioinformatics analyses were performed at the Bioinformatics Laboratory of LEEM in the facilities of INPA. The FastQC program [v.0.11.3] (Andrews, 2010) was used to analyze the quality of sequenced reads. Treatment of the 5′ and 3′ ends of the low quality reads (Q-score ≤ 20) and filtering of reads of less than 50 bp (base pairs) (≤50) were performed using the program Trimmomatic [v. 0.33] (Bolger et al., 2014).

The reassembly of the transcriptome was performed with the Trinity program [version 2.1.1] (Grabherr et al., 2011). In addition, programs that assisted Trinity to assemble the transcriptome and to calculate the abundance of transcripts were used, among them, the Bowtie2 [v. 2.2.6] (Langmead and Salzberg, 2012), RSEM [v. 1.2.19] (Li and Dewey, 2011), and R/Bioconductor packages [v. 3.1] (Gentleman et al., 2004).

### Differential Expression Analysis and Gene Annotation

The analysis of differentially expressed genes (DEGs) was performed with the R/Bioconductor package, edgeR [v. 3.8.6] (Robinson et al., 2010), with a False Discovery Rate (FDR) ≤ 0.05. After the transcript quantification, the data generated by the RSEM, served as the input to the edgeR, when fold change was calculated. The DEGs were annotated with the BLASTx program [v. 2.3.1] (Altschul et al., 1997), through consultation of the database of Uniprot/TrEMBL proteins (class Actinopterygii)^1^, with *E*-value 1.0E-5. After functional annotation, the genes were classified according to their gene ontologies (GO), using a script developed at the Multidisciplinary Support Center of the Federal University of Amazonas (CAM/UFAM).

## Results

### Physicochemical Parameters of the Waters

The physicochemical parameters are quite different, with a specific water pattern for each environmental condition (**Table 1**). Conductivity is one of the reflexes of the differences between the waters, being directly connected to the quantity of dissolved ions. It presents low conductivity in waters that are poor in ions such as the Negro River (10.5 μS/cm), with a moderate increase in the Tapajós River (17.3 μS/cm) and very high in the Solimões River (74.2 μS/cm).

**Table 1:** Physicochemical parameters of the waters of the Negro River (blackwater), Tapajós River (clearwater) and Solimões River (whitewater).

Characteristics	Types of water		
	Negro River	Tapajós River	Solimões River
	
Color	Black	Clear	White
Conductivity	10.5 ± 0.5 μS/cm^a^	17.3 ±0.3μS/cm^b^	74.2 ±0.1μS/cm^c^
Oxygen	3.1 ±0.5mg/L^a^	6.6 ±0.1mg/L^b^	2.4 ±0.2mg/L^c^
Temperature	29.5 ±0.1°C^a^	31.2 ±0.4°C^b^	28.3 ±0.1°C^c^
pH	4.6 ±0.1^a^	6.0 ±0.4^b^	6.3 ±0.1^c^
DOC^∗^	8.75 mg/L^a^	<0.4mg/L^b^	3.86mg/L^c^


*Values expressed as mean ± SD; N = 5 in each condition. Different letters indicate significant differences (P ≤ 0.05), using one-way ANOVA and Tukey test. ^∗^ DOC values from Holland et al. (2017)*.

Data recently published by Holland et al. (2017), who, for their part, carried out water collections in the same period of our collections of the specimens (July, 2015), in locations close to our collection points, corroborate with additional information on some parameters Physical-chemical properties of water. Among them, we highlight the concentration of dissolved organic carbon (DOC) for the Rio Negro of 8.75 mg/L, Solimões River (3.86 mg/L) and Tapajós River (<0.4 mg/L). We also highlight the difference concerning the pH of the respective rivers: acidic water from the Negro River with a pH of 4.6, being as low as a pH 3, the clearwater of the Tapajós River with a pH of around 6.0 and the whitewater of the Solimões River with a pH around 6.3, both close to neutrality. The blackwater is low in ions, while it has a high concentration of H^+^ (2.5 × 10^-5^ mol/L), derived from the humic and fulvic acids of the plant decomposition that occurs on the Rio Negro bed (reviewed by Val and Almeida-Val, 1995). On the other hand, the Tapajós River and the Solimões River have a low concentration of DOC, with a concentration of H^+^ equal to 1.0 × 10^-6^ mol/L and 5.0 × 10^-7^ mol/L, respectively. This represents a concentration of H^+^ in the Negro River of approximately 25 times greater than that observed in the Tapajós River and 50 times greater than that present in the Solimões River.

### Sequencing and Quality Control

Nine *T. albus* cDNA libraries were constructed, three biological replicates for each environmental condition: blackwater, clearwater, and whitewater. The RNA-Seq Sequencing in the MiSeq^®^ Platform (Illumina^®^) produced about 51.6 million paired-end reads. In the quality control and filtering of the raw data, the bases were removed from the ends of the reads with Q-Score ≤ 20 and the reads with a size of less than 50 pb were excluded, resulting in a total of 45.8 million paired-end reads that were saved. Only 11.17% of the total reads sequenced were discarded (Supplementary Table S2).

### Assembly *De Novo* and Differential Expression Analysis

**FIGURE 1:**
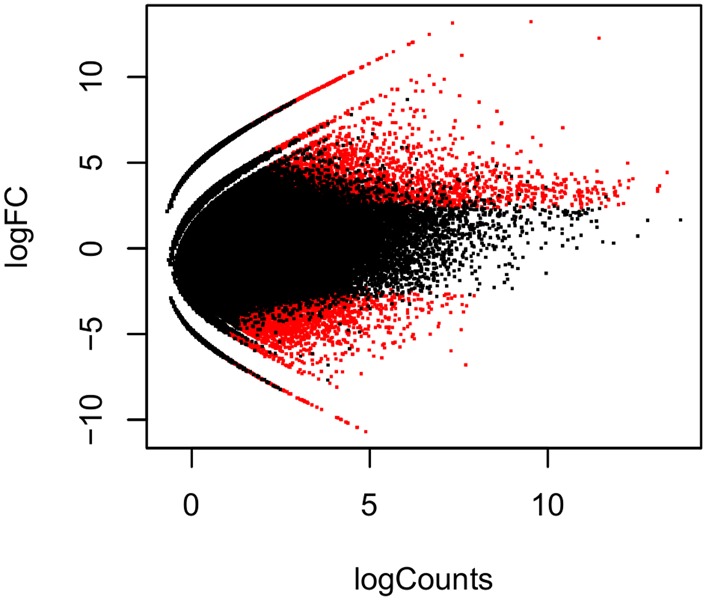
Differentially expressed genes of *Triportheus albus* in blackwater versus clearwater conditions. The data are shown in logarithmic scale (base 2), considering the fold change of the expression versus the mean of the level of expression between the conditions analyzed. The red points above zero on the y-axis represent the up-regulated transcripts, while the ones which are below zero, represent down-regulated transcripts.

The reads resulting from the pre-processing of the data were grouped and the transcriptome assembly *de novo* was performed. A total of 285,456 transcripts were assembled, using the Bruijn graph analysis (Grabherr et al., 2011), from which the contigs with average lengths of 584.93 pb were derived. The value of N50 was 751 pb, totaling 166,972,252 bases assembled. The contigs were aligned and the abundance of transcripts quantified in each environmental condition. This was done for all biological replicates. The analysis of the differential expression of *T. albus* among environmental conditions was performed using FDR ≤ 0.05. In addition to FDR, we considered fold change ≥ 2. A total of 13,754 genes were differentially expressed in the three environmental conditions. In blackwater conditions versus whitewater conditions, 3,956 DEGs were found, 265 up-regulated ones (6.7%), 3,691 down-regulated ones (93.3%). In clearwater versus whitewater conditions, only 30 differentially expressed transcripts, 2 up-regulated ones (6.7%) and 28 down-regulated ones (93.3%) were found. When it comes to blackwater versus whitewater conditions, 9,768 DEGs, 4,318 up-regulated ones (43.2%) and 5,550 down-regulated ones (56.8%) were found (**Figure 1**).

### Transcriptome Annotation of *T. albus*

Using BLASTx, with E-value of 1.0E-5, through consultation to the database Uniprot/TrEMBL (Actinopterygii class), 33,739 genes were identified. The top hits returned by a BLAST search were associated with the following species of fish: *Astyanax mexicanus* (43%), *Danio rerio* (14%), *Oncorhynchus mykiss* (7%), *Poeciliopsis prolifica* (6%), *Ictalurus punctatus* (5%) and other species (25%).

**FIGURE 2:**
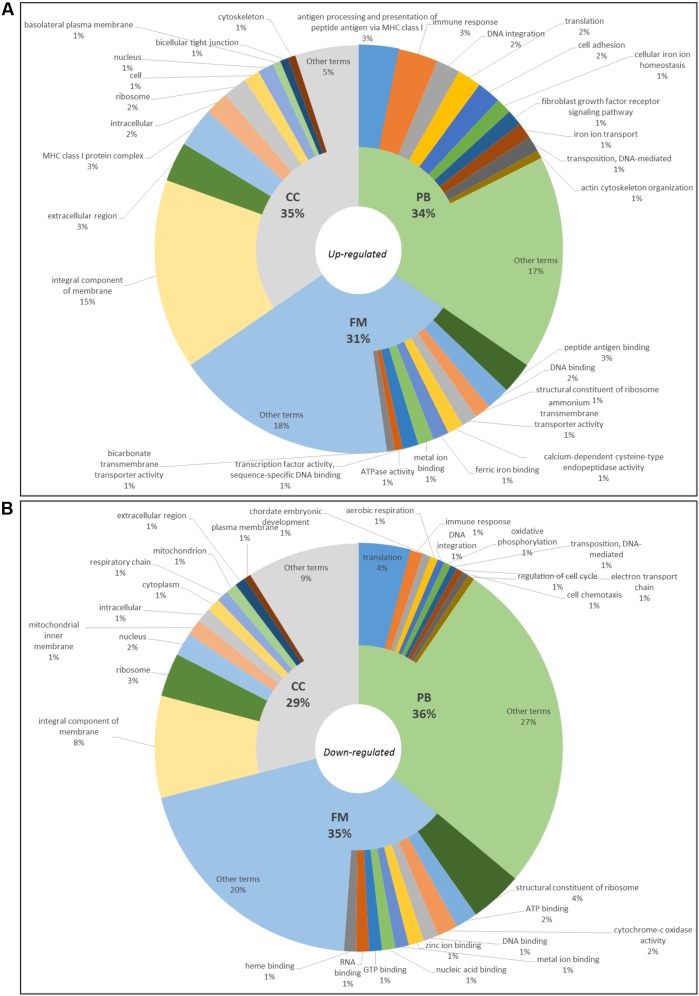
Gene ontology of top10 up-regulated **(A)** and top10 down-regulated **(B)** terms in blackwater versus whitewater conditions for *Triportheus albus.*

Differentially expressed genes were grouped according to their GO. In blackwater versus whitewater conditions, 3,206 terms were annotated. Out of these, 159 up-regulated ones (Biological Process – PB: 54, Molecular Function – FM: 50 and Cell Component – CC:55) (**Figure 2A**) and 3,047 down-regulated ones (PB: 1,100, FM: 1,064 and CC: 883) (**Figure 2B**).

**FIGURE 3:**
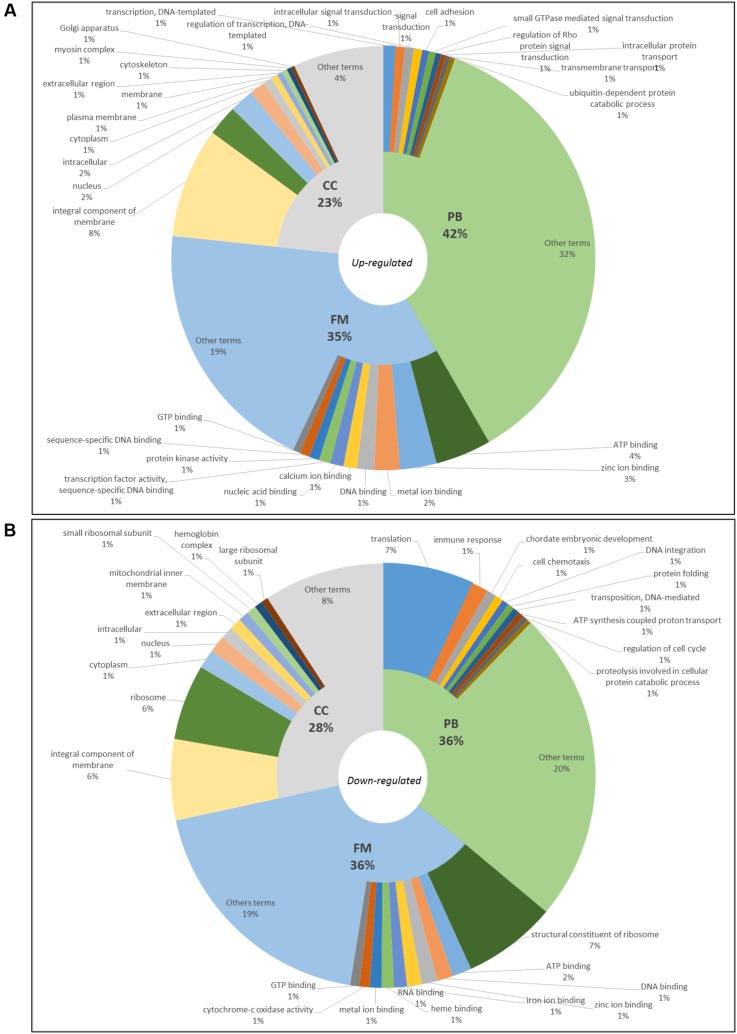
Gene ontology of top10 up-regulated **(A)** and top10 down-regulated **(B)** terms in blackwater versus clearwater conditions for *Triportheus albus.*

In blackwater versus clearwater conditions, 9,566 terms were annotated – 5,938 up-regulated ones (PB: 2,476, FM: 2,077 and CC: 1,385) (**Figure 3A**) and 3,628 down-regulated ones (PB: 2,476, FM: 2,077 and CC: 1,385) (**Figure 3B**). When it comes to clearwater versus whitewater conditions, only 21 terms were annotated (9 in PB, 9 in FM and 3 in CC, all of them were up-regulated).

### Common Terms in “Blackwater versus Clearwater” and “Blackwater versus Whitewater”

In the DEGs analysis, a greater number of transcripts expressed in the conditions of blackwater versus clearwater and blackwater versus whitewater were observed. We then verified whether such conditions share the same gene ontology terms. Thus, we located 1,551 up-regulated terms in common under both conditions. Among these, we highlight some terms such as integral component of membrane, calcium ion binding, plasma membrane, cell adhesion, cytoskeleton, myosin complex, transporter activity, ammonium transmembrane transporter activity, bicellular tight junction and ATPase activity (Supplementary Figure S1).

After that, we analyzed the genes linked to the annotated terms. We observed that the genes identified were related to a fundamental role in cellular permeability, ionic regulation and acid-base (**Table 2**). Among these, we found the claudin genes, actn4 (actinin, alpha 4), itgb3b (integrin beta) (paracellular junction and cell adhesion), rhcg1 (ammonium transporter Rh type C 1), slc9a6a (sodium/hydrogen exchanger) (NHE), ATP6V0A2 (V-type proton ATPase subunit a), Na^+^/K^+^-ATPase (sodium/potassium-transporting ATPase subunit alpha) (ionic regulation and acid-base) nr3c1 (glucocorticoid receptor), prlra (prolactin receptor a) (endocrine response) (**Table 2**).

**Table 2:** Common genes of *Triportheus albu*s in blackwater versus clearwater conditions and blackwater versus whitewater conditions, candidates involved in the response to low pH.

Contig ID	*E*-value	LogFC	Gene/Proteína	Annotation (GO)
TR35387| c0_g1_i1	5.00E-157	6.65	Ca^2+^-ATPase	Calcium ion transport (PB) and calcium-transporting
				ATPase activity (FM)
TR31771| c0_g1_i1	2.00E-11	6.33	Claudin	Bicellular tight junction (CC) and
				cell junction (CC)
TR44997| c0_g2_i1	0.00E-00	6.18	DSP	cell adhesion molecule binding (FM) and
				cell junction (CC)
TR53695| c1_g2_i1	0.00E-00	4.56	actn4	Bicellular tight junction assembly (PB) and protein
				localization to bicellular tight junction (PB)
TR49051| c2_g2_i1	0.00E-00	4.55	itgb3b	Cell-substrate junction assembly (PB) and cell adhesion
				(PB)
TR58189| c5_g2_i3	8.00E-158	4.11	Gap junction protein	Gap junction (CC) and
				cell junction (CC)
TR57503| c3_g54_i1	8.00E-48	5.86	slc26a4	Bicarbonate transport (PB) and
				chloride transport (PB)
TR39575| c0_g1_i2	0.00E-00	4.71	slc9a6a (NHE)	Sodium ion transport (PB) and hydrogen ion
				transmembrane transport (PB)
TR53402| c0_g3_i1	0.00E-00	4.04	slc4a4b	Bicarbonate transport (PB) and anion transmembrane
				transporter activity (FM)
TR42005| c0_g1_i1	8.00E-173	4.34	Na^+^/K^+^-ATPase	Sodium ion transport (PB) and sodium:potassium-
				exchanging ATPase activity (FM)
TR54587| c4_g2_i6	0.00E-00	3.82	rhcg1	Ammonium transport (PB) and ammonium
				transmembrane transporter activity (FM)
TR42033| c0_g1_i1	0.00E-00	3.16	ATP6V0A2	Ion transport (PB) and hydrogen ion transmembrane
				transporter activity (FM)
TR46875| c0_g2_i1	0.00E-00	4.73	Prlra	Prolactin signaling pathway (PB) and
				prolactin receptor activity (CC)
TR33864| c0_g2_i2	0.00E-00	3.45	nr3c1	Sodium ion homeostasis (PB) and
				regulation of ion homeostasis (PB)


## Discussion

The diversity of rivers of the Amazon basin, besides containing the largest number of species of freshwater fish, represents geographical barriers by means of its restricted water profile within each river. This requires biological adjustments of species living in these different environments (De Pinna, 2006). Many fish species live in an environment close to neutrality (Val and Almeida-Val, 1995). However, many fish species live in waters with low pH may that influence several physiological processes (Wood et al., 1998; Matsuo and Val, 2007). The species *T. albus* is one of the few species that differs from the innumerable other species of fish in the region, since it occurs in all three types of waters (blackwater, clearwater, and whitewater) in the Amazon Basin.

The work of Gibbons et al. (2017), with the fish species *Gasterosteus aculeatus*, which survives in freshwater and saltwater environments, shows an excellent approach to how the gene expression of fish responds to the most diversified environments. Although living in environments that are different from those of the species studied here, the authors observed that the environment in which the organism was being exposed directly influenced the gene expression pattern, a situation corroborated by the present study. They have also described the occurrence of phenotypic plasticity to respond to the environment to which they are exposed and to maintain the homeostasis under the prevailing conditions, a very similar situation to the one found here for *T. albus.*

To date, there is no information on the transcriptome profile of another species that is able to survive in all three types of water in the Amazon. In this context, our study is unique since we present the analysis of the transcriptome of *T. albus* living in all three types of water in the Amazon.

We observed 13,754 differentially expressed transcripts in all three environmental conditions. The DEGs were grouped by their functional categories according to the GOs (Ashburner et al., 2000). Thus, we selected the top10 terms, both up-regulated and down-regulated, from each functional category (PB, FM and CC).

In blackwater versus whitewater conditions and blackwater versus clearwater conditions, GO enriched up-regulated terms are involved mainly with membrane components, active transport of ions across the membrane, cytoskeletal/cell adhesion change, and synthesis of proteins (**Figures 2A, 3A**). These functional categories show responses to exposure to the acidic and low ion environment, as the blackwater, contrasting with whitewater and clearwater that have a pH close to neutrality (6.0 and 6.4 respectively) and are relatively richer in ions. These responses trigger cellular processes aimed at maintaining the body’s homeostasis in relation to the environment to which they are exposed. In general, the response is due to the increase in tightness of the paracellular junctions and active transport through membrane proteins (Bonga et al., 1990; Evans et al., 2005; Kumai and Perry, 2011).

In contrast, the down-regulated terms are related to reduced protein synthesis, embryonic development, cell cycle regulation, membrane components, mitochondria, and the respiratory chain (**Figures 2B, 3B**). These repressed categories may indicate a metabolic readjustment, because in addition to the essential mechanisms of the organism, it has to maintain the homeostasis under the environmental challenges to which it is exposed (Li et al., 2016). In the gene expression pattern, both in blackwater versus whitewater conditions, as in blackwater versus clearwater conditions, up-regulated genes were mainly involved with mechanisms to maintain the body’s osmotic and ionic homeostasis (**Table 2**).

Thus, some cellular mechanisms may have been deactivated/reduced to reduce energy expenditure (Li et al., 2016). Vidal-Dupiol et al. (2013) and Logan and Buckley (2015) also observed that in order to maintain the vital functions of the organism, some genes can be readjusted until the establishment of the homeostatic balance. However, future studies should analyze the reaction of repressed genes when they are exposed to the environment that is predominant in the Negro River.

In clearwater versus whitewater conditions, there was no significant difference. Only 30 differentially expressed transcripts were found and 21 terms were annotated. Such terms are related to the immune response, hemoglobin complex and ribosomal RNA. Given the small difference, considering the whole transcriptome, we concentrated on the analyses for contrasts of blackwater versus whitewater, and blackwater versus clearwater, which presented greater difference with regards to gene expression in this study.

According to the terms annotated in common, both blackwater versus whitewater conditions and blackwater versus clearwater conditions (Supplementary Figure S1) the genes of the referred terms were located, and it was found that these genes could be linked to the response to the acidic environment. Thus, we selected 14 genes involved in such a response (**Table 2**). We have grouped these genes of *T. albus* into two main response mechanisms to low pH.

The first mechanism is related to the modulation of the branchial epithelium (paracellular junctions - JPs), highlighting the genes coding claudin proteins, actn4 (actinin, alpha 4), itgb3b (integrin beta), DSP (desmoplakin), Gap junction protein, and Ca^2+^-ATPase (calcium-transporting ATPase) (**Table 2**). Several studies have shown differentiated responses in some Amazon species exposed to low pH (Gonzalez and Wilson, 2001; Matsuo and Val, 2007; Wood et al., 2014). One of the fundamental characteristics to maintain the homeostasis in the acidified waters of the Negro River is the increase of the stiffness of JPs, avoiding excessive losses of Na^+^ and Cl^-^ to the environment (Wood et al., 1998; Gonzalez et al., 2002; Matsuo and Val, 2007). Paracellular junctions represent one mode of cell-to-cell adhesion in epithelial or endothelial cell sheets, forming continuous seals around cells. The up-regulated gene claudin, together with others found in this study as DSP, itgb3b and Gap junction, encode for the primary sealing agents of JPs (Kumai and Perry, 2011; Kwong and Perry, 2013). This mechanism, supported by many genes had not been previously reported, as this mechanism is found in Amazonian cichlids, not in characids (Gonzalez and Wilson, 2001; Duarte et al., 2013).

It is worth noting that blackwater is rich in H^+^, and this could in some way affect the JPs negatively, increasing permeability, due to the important role of Ca^2+^ in cell adhesion (McDonald, 1983; Freda et al., 1991). However, blackwater have a unique characteristic that differs them from the others. It is rich in DOM with a high concentration of DOC (Walker, 1995; Johannsson et al., 2016). An important function of DOC in acidic water is the buffering capacity against damaging effects caused by low pH (Holland et al., 2012).

Campbell et al. (1997) showed that DOC can bind directly to the biological membrane of gill cells and alter the permeability of the cell membrane. In addition to that, Wood et al. (2011) pointed out an important role of DOC on JPs, stating that DOC could act with similar functions to Ca^2+^ in JPs, reducing paracellular permeability. This statement about the protective role of DOC has recently been confirmed by Duarte et al. (2016). Duarte and collaborators observed that in Ca^2+^ poor waters such as the ones in the Negro River, DOC can act with functions similar to Ca^2+^ functions to protect the integrity of JPs. Thus, our data corroborate the information of Duarte et al. (2016), showing that even in Ca^2+^ poor waters, the genes continued to be expressed so as to maintain the integrity of JPs and, consequently, to maintain the ion balance allowing survival in waters that are challenging for many aquatic species.

The second mechanism related to the up-regulated genes encompasses ion and acid-base regulation [rhcg1 (ammonium transporter Rh type C 1), slc9a6a (sodium/hydrogen exchanger) (NHE), ATP6V0A2 (V-type proton ATPase subunit a) Na^+^/K^+^-ATPase (sodium/potassium-transporting ATPase subunit alpha), slc26a4 (pedrin), slc4a4b (anion exchange protein) (**Table 2**)]. One of the essential functions to maintain homeostasis in fish is ammonia excretion (NH_3_^+^), mainly through the gills (Wood et al., 2007). Several studies have shown that the organism exposed to an acidic environment can use the NH_3_^+^ excretion function to capture Na^+^ from the environment (Kumai and Perry, 2011; Wright and Wood, 2012; Wood et al., 2014). This information was questioned taking thermodynamics into account (Parks et al., 2008). However, Kumai and Perry (2011) observed that the excretion of ammonia through the gills increased Na^+^ uptake through the NHE complex. This interaction was associated with the presence of rhcg1 glycoprotein responsible for ammonium dissociation (NH_4_^+^) in NH_3_^+^ and H^+^ creating a favorable microenvironment for the transport of NH_3_^+^ and H^+^, through rhcg1 and NHE, respectively (Wright and Wood, 2012; Ito et al., 2013). These genes were up-regulated in the analyzed speciemens *T. albus* from Rio Negro.

In addition to the rhcg1 and slc9a6a (NHE), we also observed the expression of ATP6V0A2 (H^+^-ATPase). Like the rhcg1 and NHE proteins, H^+^-ATPase has been the focus of past studies, since it is responsible for providing an intracellular environment that favors Na^+^ uptake during low pH exposure through the active H^+^ extrusion from the cell (Lin et al., 2006; Chang et al., 2009).

A favorable electrochemical environment is required for a continuous ion regulation. In this study, we also observed an up-regulation of the gene encoding Na^+^/K^+^-ATPase. This was already expected, since organisms exposed to acidic environments perform Na^+^ uptake through the mechanism described above. Thus, for the Na^+^ uptake through the NHE exchanger, Na^+^/K^+^-ATPase is vital, since intracellular Na^+^ in excess could disturb ion regulation (Marshall, 2002; Evans et al., 2005; Wood et al., 2014). In addition to the control of cellular permeability and ion regulation, it is necessary to maintain the acid-base, in view of the natural tendency of Cl^-^ loss to the acidic environment (Evans et al., 2005). We observed the up-regulated expression of the slc26a4 and slc4a4b genes. These genes are involved in the control of intracellular pH. This information corroborates Bayaa et al. (2009) and Perry et al. (2009) proposing that the uptake of Cl^-^ from the environment would occur through families of exchange proteins, such as slc26.

However, for this exchange to be possible, an intracellular HCO_3_^-^ molecule is required for the coupling of the Cl^-^/HCO_3_^-^ exchanger (Evans et al., 2005). Studies have shown that carbonic anhydrase is responsible for the supply of internal HCO_3_^-^ through the hydration of CO_2_ (Lin et al., 2008; Gilmour and Perry, 2009). In our data, we did not find the differential expression of carbonic anhydrase. However, we can verify its performance through the by-product (HCO_3_^-^), which is being used in the acid-base balance, through the gene expression for the protein slc26a4 that acts on the apical membrane of the cell by exchanging Cl^-^/HCO_3_^-^, and also for slc4a4b, a protein located in the basolateral membrane, which uses HCO_3_^-^ and Na^+^ to transport HCO_3_^-^ to the blood, keeping the internal pH balanced (Evans et al., 2005).

The mechanisms described above are essential to maintain the homeostasis of the organism when exposed to acidic environments, such as the waters of the Negro River. For these mechanisms to be triggered, many authors have reported the importance of endocrine responses in fish (as reviewed by Breves et al., 2014; Kwong et al., 2014). We highlighted the genes nr3c1 (glucocorticoid receptor) and prlra (prolactin receptor a) (**Table 2**), both involved in hormonal responses, cortisol and prolactin, respectively. According to Kwong and Perry (2013), these hormones contribute to reduce epithelial permeability, avoiding excessive losses of Na^+^ and Cl^-^ to the environment. They can also promote the reabsorption of these ions (Flik and Perry, 1989; Kelly and Wood, 2001; Kumai et al., 2012), and increase H^+^-ATPase activity (Lin and Randall, 1993).

**FIGURE 4:**
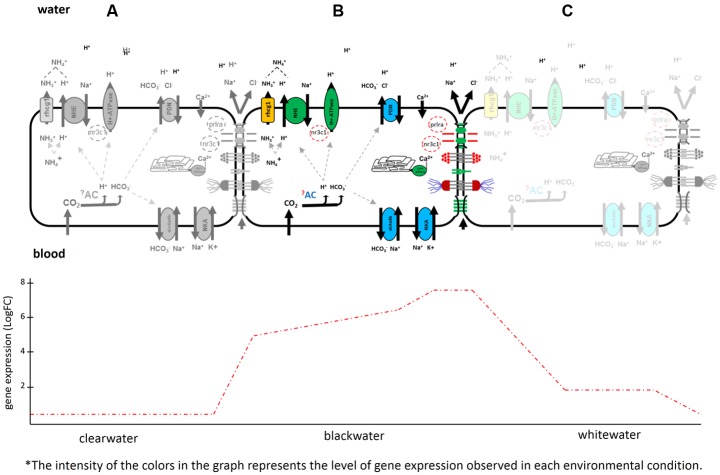
Schematic representation of phenotypic plasticity observed for *Triportheus albus.* In clearwater there was almost no expression **(A)**. Following the trajectory of the graph, there was a high differential expression of the genes found in animals exposed to blackwater **(B)**. In whitewater, the genes related to ionic regulation was reduced and the modulation of the branchial epithelium (paracellular junctions) completely disconnected **(C)**.

The ability of *T. albus* to modulate such mechanisms demonstrates a well-developed phenotypic plasticity system for this species. The potential mechanisms described here, based on the gene expression, demonstrate the potential of *T. albus* to survive the challenges presented by the different types of water in the Amazon basin. Depending on the environmental condition to which it is exposed, the genotype of this species can be differentially transcribed (**Figure 4**). The review by Schneider and Meyer (2017) highlights the mechanisms of differential expression. Analyzing **Figure 4**, in clearwaters (A) the activation level of ion and acid-base response mechanisms (genes coding for respective proteins involved) are almost null when compared to animals collected in blackwaters (B). In blackwater animals, every expressed mechanism for the maintenance of ion homeostasis is well characterized. In whitewater animals, these mechanisms are being expressed in an intermediate form (C), when compared to clearwater and blackwater conditions.

**FIGURE 5:**
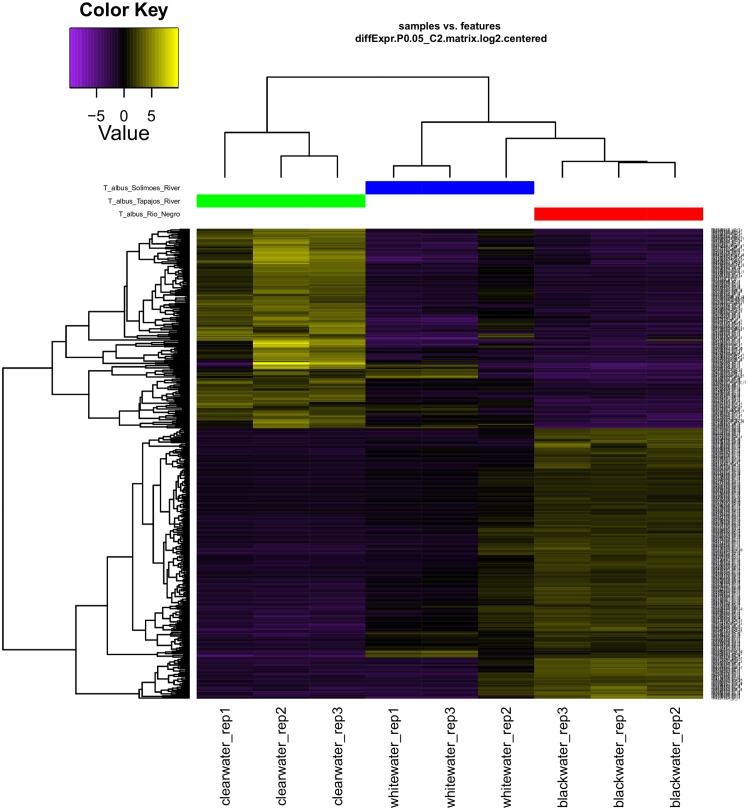
Expression patterns (heatmap) and hierarchical clusters of the genes of *Triportheus albus* specimens differentially expressed in different habitats (blackwater, whitewater and clearwater). Dendrogram transcription patterns were estimated only for differentially expressed genes. Bar colors reflect expression levels of the gene, black (low), purple (down-regulated), and yellow (up-regulated).

Physiological responses of Amazonian fish have been better understood over the years (Matsuo and Val, 2007; Duarte et al., 2013; Wood et al., 2014). The use of new techniques of molecular biology and bioinformatics resources, has increased the knowledge of how some species of fish respond to adverse environmental conditions (Lemgruber et al., 2013; Jesus et al., 2016; Prado-Lima and Val, 2016). In this context, we could observe in the present study that the *T. albus* species responds differently according to the exposed environment (**Figure 5**). When analyzing the heatmap graph, we verified two clusters of differential gene responses, related to the two extremes of aquatic environments.

Although literature emphasizes that clearwater is classified as intermediate between blackwater and whitewater (Sioli, 1984; Duncan and Fernandes, 2010), we can observe that *T. albus* presents a larger set of adjustments in gene expression in the Tapajós River (clearwater) and in the Negro River (blackwater). Thus, through the gene response observed in this species, we can conclude that clearwater and blackwater are two distinct extremes, and they present a greater environmental challenge for the survival of the organism. On the other hand, we verified that the whitewater was the one that presented an intermediate level, requiring less quantitative adjustment of the gene response to the environment to which it was exposed. Therefore, the gene expression pattern observed for *T. albus* suggests that this species presents phenotypic plasticity to live in the three main types of water in the Amazon.

## Conclusion

The Negro River is the most critical environment for the survival of many aquatic species in the Amazon basin, due to its high acidity and low ion concentration. As this river system harbors more 1,000 species, it is possible that more species present similar phenotypic plasticity as *T. albus* that showed two main mechanisms that allow survival in Amazonian aquatic environments, including those with low pH. The first mechanism is the control of genes in paracellular junctions, such as claudin, actn4, itgb3b, DSP that are involved in the process of maintaining the paracellular permeability control and, consequently, the loss of Na^+^ and Cl^-^ ions to the environment. This characteristic that until then had been observed only in Amazon cichlids, was well developed in *T. albus*, a species of characid.

The second mechanism was attributed to the ability of ionic and acid-base regulation developed by this species. We observed high expression of the genes involved in Na^+^ uptake, where excretion of NH_3_^-^ through the rhcg1 protein somehow favors Na^+^ uptake through the NHE exchanger, in addition to the H^+^-ATPase and the Na^+^/K^+^-ATPase pump. We also find the prlra and nr3c1 genes, responsible for triggering the two mechanisms described above. Therefore, we could verify that the species *T. albus* presents phenotypic plasticity with mechanisms that confer abilities to survive in environments considered critical for many species. We suggest that the species *T. albus* is a good candidate for future studies involving ion and acid-base regulation processes, as well as to analyze the activities of the respective enzymes involved in these processes.

## Author Contributions

JA, AG, and AV designed the work. JA and AG analyzed and interpretation of the data. JA and AV drafted the work. All the authors approve the final version.

## Conflict of Interest Statement

The authors declare that the research was conducted in the absence of any commercial or financial relationships that could be construed as a potential conflict of interest.

## Footnotes

1 www.uniprot.org
