# Supplementary material for: Triportheus albus Cope, 1872 in the Blackwater, Clearwater, and Whitewater of the Amazon: A Case of Phenotypic Plasticity?
Source: Front Genet. 2017 Aug 31;8:114. doi: 10.3389/fgene.2017.00114 (PMC5583242; doi:10.3389/fgene.2017.00114)
Supplement: Supplementary file 1 [file Data_Sheet_1.doc]

Supplementary Material

# *Triportheus albus* Cope, 1872 in the Blackwater, Clearwater and Whitewater of the Amazon: A Case of Phenotypic Plasticity?

José Deney Alves de Araújo^1,2 *^, Andrea Ghelfi^3^, Adalberto Luis Val^1^

(1) National Institute for Research in the Amazon, Laboratory of Ecophysiology and Molecular Evolution, Manaus, Amazonas, Brazil

(2) Federal University of Amazonas, Manaus, Amazonas, Brazil

(3) Kazusa DNA Research Institute, Kisarazu, Chiba, Japan

* deneyaraujo@gmail.com

# Supplementary Table

**Table 1S.** Length (cm) and mass (g) of the fish collected in blackwater, clearwater and whitewater.

| **Conditions** | **Length (cm)** | **mass (g)** |
| --- | --- | --- |
| Blackwater | 25.2±2.3 | 13.7±0.7 |
| Clearwater | 27.6±8.9 | 13.2±1.2 |
| Whitewater | 29.6±1.0 | 13.5±0.8 |

Values expressed as mean ± SD; N = 3 in each condition.

# Supplementary Table

**Table 2S.** Statistical results of raw and preprocessed sequences.

| **Reads** | **Blackwater** | **Clearwater** | **Whitewater** |
| --- | --- | --- | --- |
| Raw reads | 6.349.720 (100 %) | 27.340.067 (100 %) | 17.932.268 (100 %) |
| Cut out reads | 535.841 (8,43 %) | 3.255.075 (11,9 %) | 1.980.282 (11,04 %) |
| Clean reads | 5.813.879 (91,56 %) | 24.084.992 (88,09 %) | 15.951.986 (88,95 %) |
| CG | 47,66 % | 48,0 % | 48,66 % |

# Supplementary Figure


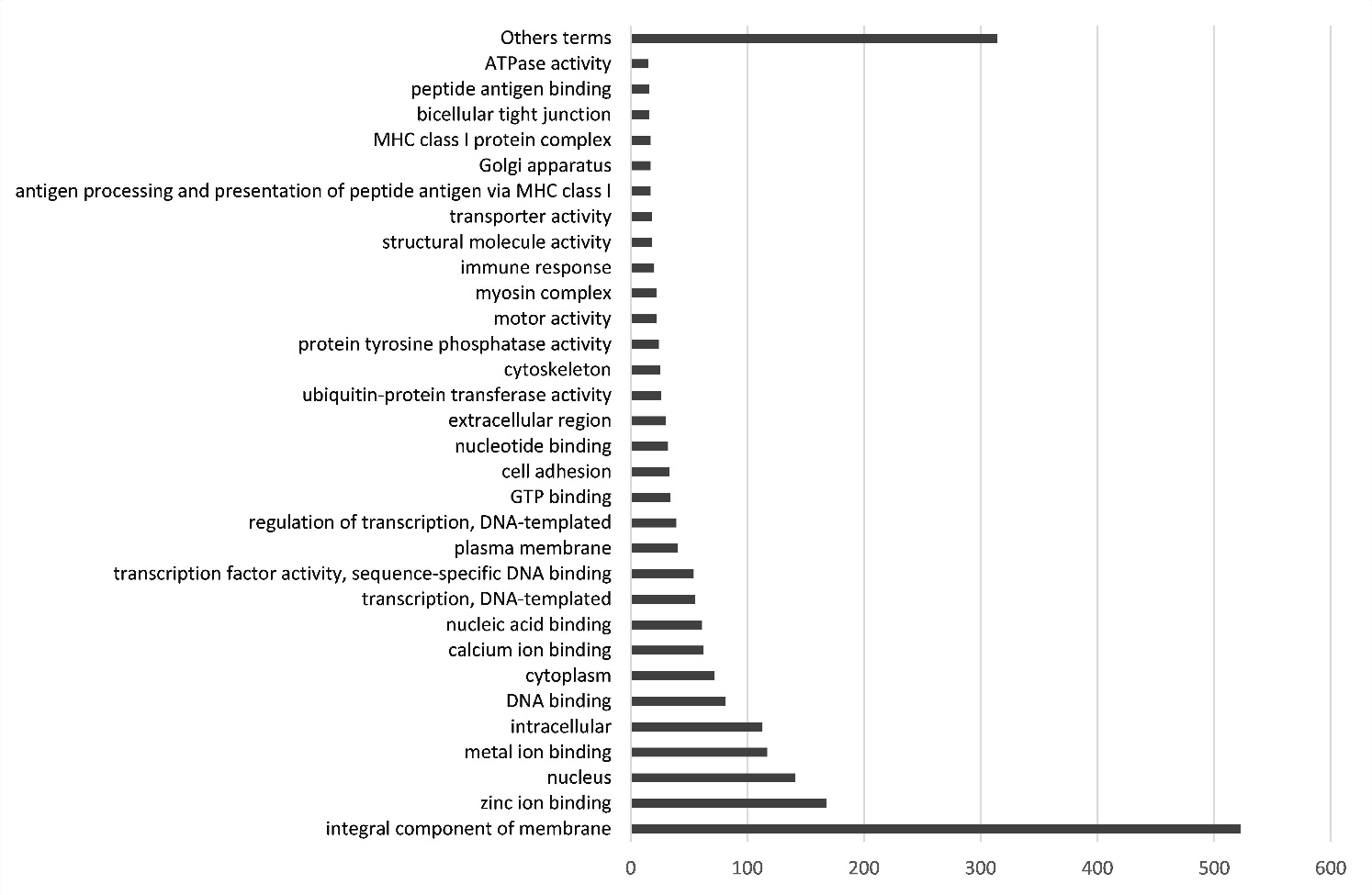


**Figure 1S.** Common terms for *Triportheus albus* in blackwater versus clearwater conditions (y axis). Number of terms is indicated on x-axis.
